# Comparative genomics reveals key adaptive mechanisms in pathogen host-niche specialization

**DOI:** 10.3389/fmicb.2025.1543610

**Published:** 2025-06-06

**Authors:** Menglu Zhang, Longxi Han, Caizhi Liao, Weiheng Su, Chunlai Jiang

**Affiliations:** ^1^National Engineering Laboratory for AIDS Vaccine, School of Life Science, Jilin University, Changchun, China; ^2^Department of General Surgery, The First Hospital of Tsinghua University, Beijing, China; ^3^Clinical Laboratory, The Fourth People’s Hospital of Jinan, Jinan, China; ^4^Key Laboratory for Molecular Enzymology and Engineering, The Ministry of Education, Jilin University, Changchun, China

**Keywords:** bacterial adaptation, comparative genomics, host-pathogen interactions, ecological niches, antibiotic resistance, virulence factors, *hypB* gene

## Abstract

**Introduction:**

Understanding the key factors that enable bacterial pathogens to adapt to new hosts is crucial, as host-microbe interactions not only influence host health but also drive bacterial genome diversification, thereby enhancing pathogen survival in various ecological niches.

**Methods:**

We conducted a comparative genomic analysis of 4,366 high-quality bacterial genomes isolated from various hosts and environments. Bioinformatics databases and machine learning approaches were used to identify genomic differences in functional categories, virulence factors, and antibiotic resistance genes across different ecological niches.

**Results:**

Significant variability in bacterial adaptive strategies was observed. Human-associated bacteria, particularly from the phylum *Pseudomonadota*, exhibited higher detection rates of carbohydrate-active enzyme genes and virulence factors related to immune modulation and adhesion, indicating co-evolution with the human host. In contrast, bacteria from environmental sources, particularly those from the phyla *Bacillota* and *Actinomycetota*, showed greater enrichment in genes related to metabolism and transcriptional regulation, highlighting their high adaptability to diverse environments. Bacteria from clinical settings had higher detection rates of antibiotic resistance genes, particularly those related to fluoroquinolone resistance. Animal hosts were identified as important reservoirs of resistance genes. Key host-specific bacterial genes, such as *hypB*, were found to potentially play crucial roles in regulating metabolism and immune adaptation in human-associated bacteria.

**Discussion:**

These findings highlight niche-specific genomic features and adaptive mechanisms of bacterial pathogens. This study provides valuable insights into the genetic basis of host-pathogen interactions and offers evidence to inform pathogen transmission control, infection management, and antibiotic stewardship.

## Introduction

Bacterial pathogens have the exceptional capacity to colonize and infect a wide range of hosts, including animals, plants, and insects, across diverse ecological niches (Baquero et al., 2021; van Baarlen et al., 2007). This often involves host switching, which underlines the complex interdependencies within ecosystems as highlighted by the WHO’s One Health approach. This approach is crucial as it integrates human, animal, and environmental health, acknowledging that the health of each is interconnected and dependent on the other (Hernando-Amado et al., 2019). The emergence of new infectious diseases and the widespread use of antibiotics have intensified public health challenges, emphasizing the need for a holistic view in tackling these issues (Barber and Fitzgerald, 2024). Therefore, identifying the factors that control the ability of pathogens to adapt to new host species is a critical research priority (Barber and Fitzgerald, 2024). Understanding the genetic basis and molecular mechanisms that enable these pathogens to adapt to different environments and hosts is essential for developing targeted treatment and prevention strategies (Sheppard et al., 2018).

The genomic diversity of pathogens plays crucial roles in their adaptability (Toft and Andersson, 2010). DNA mutation and repair, as well as horizontal gene transfer, are key genetic mechanisms of bacterial evolution, enabling pathogens to survive and proliferate in various environments (Morley et al., 2015; Ochman et al., 2000). Ample evidence suggests that the host environment has a profound effect on the bacterial genome, leading to genetic differentiation. For example, the ability of pathogens like *Vibrio parahaemolyticus* to exhibit distinct ecotypes in different environments, and the transition of *Pseudomonas aeruginosa* from environmental niches to human hosts, underscores the relevance of understanding ecological influences on bacterial behavior (Cui et al., 2015; de Bentzmann and Plesiat, 2011). Bacteria adapt to their host environment primarily through gene acquisition and gene loss (Sheppard et al., 2018). Horizontal gene transfer is common among host-associated microbiota (Soucy et al., 2015). A notable example is *Staphylococcus aureus,* which has acquired a variety of host-specific genes through this process. These include immune evasion factors in equine hosts, methicillin resistance determinants in human-associated strains, heavy metal resistance genes in porcine hosts, and lactose metabolism genes in strains adapted to dairy cattle (Richardson et al., 2018). On the other hand, gene loss also represents a critical adaptive strategy (Albalat and Cañestro, 2016). *Mycoplasma genitalium* has undergone extensive genome reduction, including the loss of genes involved in amino acid biosynthesis and carbohydrate metabolism (Fraser et al., 1995), enabling the bacterium to reallocate limited resources toward maintaining a mutualistic relationship with its host. However, identifying the specific genes responsible for niche adaptation remains a challenge, requiring robust comparative approaches to differentiate core genome content from niche-specific adaptations.

Recent advances in whole-genome sequencing and comparative genomics have provided powerful tools and new insights into the genetic basis of niche adaptation in human pathogens (Brynildsrud et al., 2016; McAdam et al., 2014). By integrating genome-wide association studies (GWAS) with population genomics, researchers can identify genes associated with specific ecological niches or host-specific adaptations, offering a deeper understanding of the processes of adaptation, diversification, and reductive evolution during host adaptation (Keen et al., 2021; Toft and Andersson, 2010; Wang et al., 2022). Combining the identification of host-adaptive genetic traits with functional analyses can reveal the biological mechanisms underlying the colonization of new host species, including key host-pathogen interactions that may represent novel therapeutic targets (Sheppard et al., 2018).

In this study, we investigated the genomic differences between human pathogens isolated from various hosts (humans and animals) and environmental sources to identify niche-associated signature genes. Using 4,366 high-quality, non-redundant pathogen genomes, we conducted comparative genomic analyses with multiple bioinformatics databases (COG, dbCAN, VFDB, CARD) to explore functional and pathogenic variations across ecological niches. This study reveals the differential strategies employed by various phyla of human-associated pathogenic microorganisms to adapt to the human host. Specifically, *Pseudomonadota* utilize a gene acquisition strategy, while *Actinomycetota* and certain *Bacillota* employ genome reduction as an adaptive mechanism. Additionally, we identified animal-derived pathogens as significant reservoirs of virulence and antibiotic resistance genes. Adaptive genes linked to specific niches were identified using Scoary, and machine learning algorithms were applied to enhance predictive accuracy. We also discovered potential human host-specific signature genes, such as *hypB*. Our findings provide insights into the genetic basis of pathogen adaptation, shedding light on how pathogens evolve under niche-specific selection pressures. This study offers a framework for future research on pathogen evolution, host-pathogen interactions, and the development of new antimicrobial strategies.

## Materials and methods

### Collecting genome datasets for comparative genomic analysis

We obtained metadata information for 1,166,418 human pathogens from the gcPathogen database^1^ (Guo et al., 2024). To construct a high-quality and non-redundant genome collection, we implemented stringent quality control procedures (Levy et al., 2017). First, we performed an initial quality control based on the metadata information. Given the sufficient data volume, we excluded sequences assembled at the contig level. We retained genome sequences with N50 ≥50,000 bp and those that passed CheckM evaluation with completeness ≥95% and contamination <5%. Next, we removed bacterial genomes with unclear source information. Based on isolation source and host information, we annotated bacterial genomes with ecological niche labels (human, animal, environment), which served as labels for subsequent analyses.

The classification into ecological niche labels was based on detailed metadata annotations of isolation sources and host information. Specifically:

1) Human: Genomes were categorized as “human” if the isolation source explicitly indicated a human host or clinical sample, such as blood, urine, stool, or other human-derived tissues and microbiomes. These samples were primarily associated with human diseases or health-related studies.
2) Animal: Genomes were assigned to the “animal” category if the metadata indicated isolation from non-human animals, including domestic livestock (e.g., cattle, swine, poultry) and wildlife (e.g., deer, birds). This category also accounted for samples from animal infections or healthy microbiota surveys.
3) Environment: Genomes were labeled as “environment” if isolated from natural settings, including water, soil, air, or surfaces not directly linked to specific hosts. This included environmental surveillance samples, such as those collected from agricultural environments.

This classification system was designed to reflect the ecological and functional contexts of the pathogens, enabling a detailed analysis of their adaptation to different ecological niches. In total, we downloaded 86,135 bacterial genomes for further analysis.

Subsequently, we calculated genomic distances using Mash and clustered the data through Markov clustering, removing bacterial genomes with genomic distances ≤0.01. Finally, we identified and excluded four genome sequences where the assigned taxonomic information differed from their phylogenetic placement. As a result, we retained 4,366 pathogen genome sequences for subsequent comparative genomics analyses.

### Construction of phylogenetic tree of bacteria

To construct the phylogenetic tree, we first retrieved 31 universal single-copy genes from each genome using AMPHORA2 (Kerepesi et al., 2014). For each marker gene, multiple sequence alignments were generated using Muscle v5.1 (Edgar, 2022). Finally, we concatenated the 31 alignments into a single comprehensive alignment and constructed a maximum likelihood tree using FastTree v2.1.11 (Price et al., 2009), with visualization performed through iTOL.^2^

To compare the genomic differences among bacteria from different ecological niches within the same ancestral clade and to identify characteristic genes, we first converted the phylogenetic tree into an evolutionary distance matrix using the R package ape. Subsequently, we performed *k*-medoids clustering using the pam function from the R cluster package. To determine an appropriate number of clusters, we calculated the average silhouette coefficient for all clusters across a range of k values (*k* = 1 to *k* = 10). This range was chosen to balance resolution and interpretability: selecting too many clusters (i.e., very fine-grained populations) could complicate comparisons across ecological niches and reduce the generalizability of the analysis. The maximum average silhouette coefficient of 0.63 was observed at *k* = 8, which was selected as the optimal clustering solution.

### Functional categorization, pathogenic mechanism annotation, and enrichment analysis

For the functional categorization of bacterial genomes, we initially predicted open reading frames (ORFs) using Prokka v1.14.6 (Seemann, 2014). Subsequently, RPS-BLAST (BLAST v2.15.0) was employed to map the predicted ORFs to the Cluster of Orthologous Groups (COG) database, applying an e-value threshold of 0.01 and a minimum coverage of 70%. To annotate carbohydrate-active enzyme genes, dbCAN2 was used to map the ORFs to the CAZy database (Zhang et al., 2018), with filtering based on the parameter hmm_eval 1e-5 (Rao et al., 2023), retaining only the annotations produced by the HMMER tool.

In order to further investigate the pathogenic mechanisms of bacterial genomes, we utilized ABRicate v1.0.1 to map bacterial genomes to the VFDB database for the identification of virulence genes, using default parameters (Liu et al., 2022). Subsequently, antibiotic resistance genes were identified using RGI and the CARD database (Alcock et al., 2023), retaining only resistance genes with an identity score of ≥0.7.

In investigating the intergroup differences in functional categories and pathogenic mechanisms across bacterial genomes with distinct labels, we first determined the median copy number of genes in each functional category across bacterial genomes in each group. Subsequently, we calculated the fold change between groups and employed the Mann–Whitney *U* test to assess the statistical significance of these intergroup differences. To control for the false positive rate introduced by multiple testing, we applied false discovery rate (FDR) correction, considering a *q*-value of less than 0.05 as the threshold for statistical significance. Based on these analytical results, we further performed enrichment analysis to identify the enrichment of specific functional category genes and pathogenicity-related genes across different bacterial populations.

### Identification of niche-associated gene clusters

Based on the clustering results of functional categories, virulence, and resistance-related genes, we utilized Scoary for the preliminary identification of niche-associated signature genes. Gene clusters with a Benjamini–Hochberg FDR-adjusted *p*-value of less than 0.05 were considered enriched (Brynildsrud et al., 2016). Following this, we employed the “randomForest” package in R to construct a random forest model, performing five-fold cross-validation to further filter niche-associated signature genes. To obtain more robust results, we also applied LASSO regression using the “glmnet” package in R, with 10-fold cross-validation to select the most representative signature genes.

Finally, the receiver operating characteristic (ROC) curve visualizes a binary classifier’s diagnostic ability by plotting the true positive rate against the false positive rate at different thresholds. The area under the curve (AUC) reflects the classifier’s overall performance, with values closer to 1 indicating better discrimination (de Hond et al., 2022). We utilized the roc function from the R package “pROC” to plot the ROC curve and calculate the AUC value in order to evaluate the classification performance of the feature genes.

### Data availability

Data used in this study are available from the NCBI database, with metadata from the gcPathogen database. FTP links for genome sequences are in Supplementary Table S1. The code is available at https://github.com/zhangml1112/genomic-signatures-of-host-adaptation.git.

## Results

### Genomic dataset construction and taxonomic classification of human pathogenic bacteria

**Figure 1:**
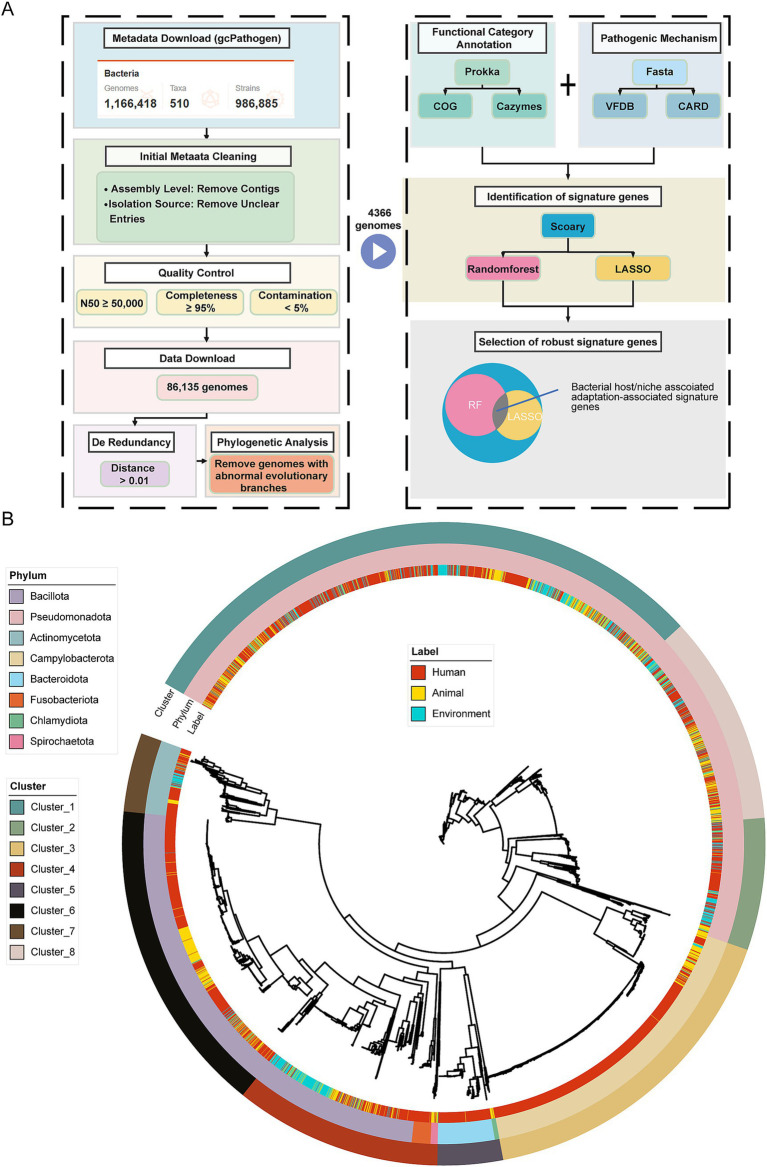
Research design and genome dataset used for comparative genomic analysis. **(A)** A schematic representation of the approach used to identify bacterial host niches adaptation-associated marker genes. As described in the Materials and Methods, we curated a genome dataset by first filtering based on genome metadata, followed by a series of quality control steps, for comparative genomic analysis. This resulted in a high-quality, non-redundant dataset comprising 4,366 bacterial genomes. The genomes were subsequently annotated using multiple databases, including the COG, CAZymes, VFDB, and CARD. Finally, host-niches-associated signature genes were identified using Scoary in conjunction with two machine learning approaches (Random Forest and LASSO regression). **(B)** Maximum-likelihood phylogenetic tree constructed from 31 conserved marker genes across the 4,366 bacterial genomes. The three rings of annotations represent, from the inside to the outside: host niches of bacterial isolation, phylum-level taxonomic classification, and clusters generated using k-medoids clustering.

We extracted metadata for 1,166,418 human pathogenic bacterial genome sequences from the gcPathogen database (see text footnote 1). This dataset includes detailed information on isolation environments and essential quality control data, providing a foundation for our subsequent analysis. As described in the Materials and Methods section, we filtered the data to remove low-quality assemblies and redundant genome information, retaining 4,366 high-quality, non-redundant sequences (Figure 1A). These sequences represent eight bacterial phyla, and based on host and isolation source information from the gcPathogen database, we classified the ecological niches of these genomes into three categories: human, animal, and environment (Table 1; Supplementary Table S1). The classification criteria, as outlined in the Materials and Methods section, ensured that each genome was assigned to a specific niche according to its isolation source and host information. This approach highlights the distinct ecological contexts of the sampled bacteria, laying the groundwork for investigating patterns of genomic variation and potential ecological adaptations.

**Table 1:** Distribution of genomes across ecological niches and taxonomic clusters (clusters are presented in the order of analysis priority: 1, 2, 8, 4, 7, followed by less-represented clusters 3, 5, and 6).

Taxa	Phylum	Human	Animal	Environment
Cluster 1	*Pseudomonadota*	725	330	286
Cluster 2	*Pseudomonadota*	182	27	89
Cluster 8	*Pseudomonadota*	219	157	97
Cluster 4	*Bacillota/Fusobacteriota/Spirochaetota*	202	102	163
Cluster 7	*Actinomycetota*	124	21	32
Cluster 3	*Campylobacterota*	663	79	8
Cluster 5	*Bacteroidota/Chlamydiota*	138	11	0
Cluster 6	*Bacillota*	506	189	17

For phylogenetically relevant comparative genomic analyses, we clustered the 4,366 genomes into eight taxonomic clusters (Clusters) via k-medoids, and we conducted subsequent comparative genomics analyses within the same clusters. We adopted this approach to reduce phylogenetic effects and ensure that differences in the studies only related to the ecological niche of the bacterial hosts we studied (Figure 1B and Table 1). Among them, five clusters (1, 2, 8, 4, and 7) showed a relatively balanced distribution across the three ecological niches, constituting a core subset of the gene-phenotype correlation analysis across ecological niches. By comparing these strains that are stably present across ecological niches, the objective is to identify which genetic modules or genes are associated with bacterial colonization in human hosts.

Clusters 1, 2, and 8 belong to the *Pseudomonadota* and are given priority in our analysis due to their sufficient representation and distinct taxonomic characteristics. Specifically, Cluster 1 includes representative genera such as *Enterobacter*, *Escherichia*, and *Vibrio*; Cluster 2 includes *Stenotrophomonas*, *Burkholderia*, *Legionella*, and others; and Cluster 8 includes *Acinetobacter* and *Pseudomonas*.

Following these clusters, Cluster 4 and Cluster 7 were also analyzed due to their sufficient representation, although they do not belong to *Pseudomonadota.* Cluster 4 includes representative species from three phyla—*Bacillota, Fusobacteriota, and Spirochaetota*—and genera such as *Bacillus*, *Staphylococcus*, *Listeria*, *Fusobacterium*, and *Borrelia*. In contrast, Cluster 7 consists of genera from the phylum *Actinomycetota*, including *Micrococcus, Gardnerella*, *Corynebacterium*, *Actinomyces*, and *Mycobacterium*.

The remaining three clusters (3, 5, and 6) are excluded from the core analysis due to limitations in their source distribution. Specifically, the number of genomes from environmental or animal sources in these clusters was fewer than 17, rendering them insufficient for Scoary comparative analysis. Cluster 3 belongs to *Campylobacterota* and includes representative genera such as *Helicobacter* and *Campylobacter*. Representative genera from *Bacteroidota* and *Chlamydiota* were classified into Cluster 5, mainly including *Bacteroides*, *Prevotella*, and *Chlamydia*. Cluster 6 mainly consists of the genera *Streptococcus* and *Enterococcus* from the phylum *Bacillota*.

In summary, this study contains two main analytical focuses: first, we focused on the five Clusters with sufficient sample sizes, of which Clusters 1, 2, and 8 are from the *Pseudomonadota*, Cluster 4 contains mainly *Bacillota*, and Cluster 7 represents the *Actinomycetota*, whereas Clusters 3, 5, and 6, with insufficient sample sizes, were not included in the analyses. Second, we focused on characterizing bacterial genes involved in human host adaptation by comparing the genomes of these bacteria adapted to different ecological niches.

### Host-dependent distribution of functional gene categories

Based on the rigorously screened collection of genomes, which we functionally annotated using the COG (Cluster of Orthologous Groups) and CAZymes databases, we compared genomes with a common ancestor (from the same cluster) isolated from human, animal, and environmental sources. This comparison revealed distinct patterns of gene functional enrichment, reflecting different adaptation strategies to specific host ecological niches.

**Figure 2:**
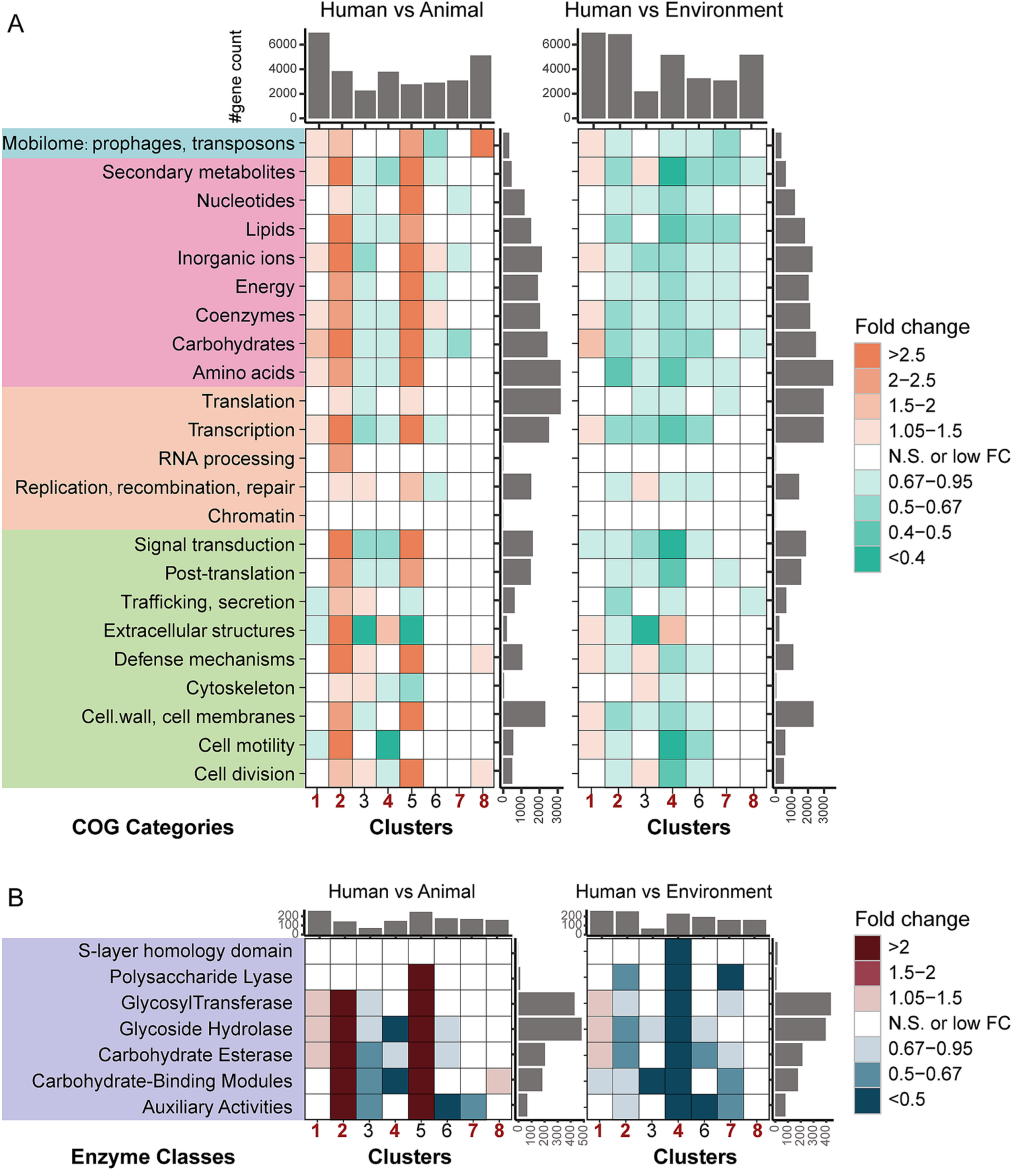
Fold-change differences in functional categories among bacterial genomes from different hosts/niches within the same taxa. **(A)** Fold-change differences in COG categories based on gene counts. The colored boxes in the figure represent different COG (Clusters of Orthologous Groups) categories, with each color corresponding to a specific class. From top to bottom, the classes are as follows: an unclassified category (X), metabolism, information storage and processing, and cellular processes and signaling. **(B)** Fold-change differences in CAZymes families based on gene counts. The heatmap illustrates levels of enrichment and depletion, determined using the Mann–Whitney *U* test. Colored cells indicate statistically significant differences (*p* < 0.05, after FDR correction), with distinct colors representing varying fold changes. “N.S.” denotes non-significant differences. The bar plots on the sides represent the summed medians of gene counts for the respective row/column groups. Full COG category names are presented in Supplementary Table S2.

Utilizing 23 broad gene categories annotated to the COG, we evaluated the enrichment or depletion of certain gene categories across the whole genomes of human pathogens isolated from human, animal, and environmental sources (Figure 2A). Enrichment analysis was statistically validated using the Mann–Whitney *U* test. Our results indicate significant differences in functional gene categories among bacteria from different sources, further highlighting the profound impact of host niches on the evolution of pathogens. Bacteria in different taxa employ diverse strategies to cope with the specific environmental pressures of these niches, exhibiting unique adaptive advantages.

Compared to animal-associated bacteria, human-associated bacteria from the *Pseudomonadota* (Clusters 1, 2, and 8) exhibited significant enrichment of genes in multiple functional categories, with genes related to “Mobilome: prophages, transposons” being significantly enriched in all three clusters (Figure 2A and Supplementary Table S2). In Cluster 1, the gene enrichment in human-associated bacteria involved several functional categories, including four metabolic pathways: “Carbohydrate transport and metabolism,” “Coenzyme transport and metabolism,” “Inorganic ion transport and metabolism” and “Secondary metabolites biosyntheisis, transport and catabolism,” as well as genes related to “Mobilome: prophages, transposons” and “Transcription.” Meanwhile, in Clusters 2 and 8, the abundance of functional genes showed a clear gradient distribution: environmental > human > animal sources. In contrast to the *Pseudomonadota*, functional genes in *Bacillota* (Cluster 4) and *Actinomycetota* (Cluster 7) were more enriched in animal and environmental sources (environmental > animal > human). Specifically, in Cluster 4, human-derived bacteria showed significant enrichment of genes related to “Extracellular structures” while in Cluster 7, human-derived bacteria exhibited significant depletion of genes related to “Inorganic ion transport and metabolism.”

We also employed dbCAN2 to analyze the carbohydrate utilization potential of each genome (Figure 2B). In total, we identified 534 carbohydrate enzyme genes, which mainly belonged to six major carbohydrate families (GT, glycosyltransferases; GH, glycoside hydrolases; CE, carbohydrate esterases; CBM, carbohydrate-binding modules; AA, auxiliary activities; PL, polysaccharide lyases) and one less common module (SLH, S-layer homology domain). At the whole-genome level, we observed results consistent with the COG annotation, indicating that host niche and taxonomy have profound effects on bacterial adaptation. Overall, human-derived bacteria in the *Pseudomonadota* (Clusters 1, 2, and 8) possess significantly more carbohydrate-active enzyme genes than animal-derived bacteria. In contrast, *Bacillota* (Cluster 4) and *Actinomycetota* (Cluster 7) show the opposite trend, with animal-derived bacteria having more carbohydrate-active enzyme genes than human-derived bacteria. Furthermore, except for Cluster 1, human-derived bacteria in all other clusters typically contain fewer carbohydrate-active enzyme genes compared to environmental isolates.

Specifically, as shown in Figure 2B, human-derived bacteria in Cluster 1 contain significantly more genes from families such as GT, GH, and CE. In Cluster 2, human-derived bacteria are significantly enriched in genes from the GT, GH, CE, CBM, and AA families compared to animal-derived bacteria. In Cluster 8, human-derived bacteria show significant enrichment only in genes from the CBM family compared to animal-derived bacteria. In contrast, in Cluster 4 and Cluster 7, human-derived bacteria exhibit a significant reduction in carbohydrate-active enzyme genes. In Cluster 4, human-derived bacteria show depletion of genes from three carbohydrate-active enzyme families (GH, CE, and CBM), while in Cluster 7, a depletion of genes from one family (AA) is observed in human-derived bacteria. Interestingly, environmental bacteria in Cluster 4 are significantly enriched in SLH domain genes, which are specifically found in bacterial surface proteins, a feature not observed in other clusters. These findings not only highlight the diversity of bacterial carbohydrate metabolism pathways but also emphasize the impact of host origin on bacterial adaptation mechanisms.

In conclusion, based on the functional category analysis, we observed differentiated human host adaptation traits in human-derived bacteria across different phyla. In *Pseudomonadota*, human-derived strains are significantly enriched in genes related to carbohydrate metabolism enzymes and mobile genetic elements, reflecting their strategy of adapting to the human environment by acquiring new functions. In contrast, human-derived strains in *Bacillota* and *Actinomycetota* exhibit characteristics of genomic streamlining, with lower functional gene abundance compared to their animal-derived counterparts. Additionally, we found that most human-derived bacteria have fewer functional genes compared to environmental isolates. These findings suggest that the process of microbial adaptation to host environments is a phylum-specific functional reorganization process.

### Host-specific distribution of virulence factor clusters

In order to explore the relationship between host niches and pathogenic mechanisms, we first annotated virulence genes in 4,366 genome sequences using the VFDB database, identifying 595 virulence factors categorized into 13 virulence factor clusters.

**Figure 3:**
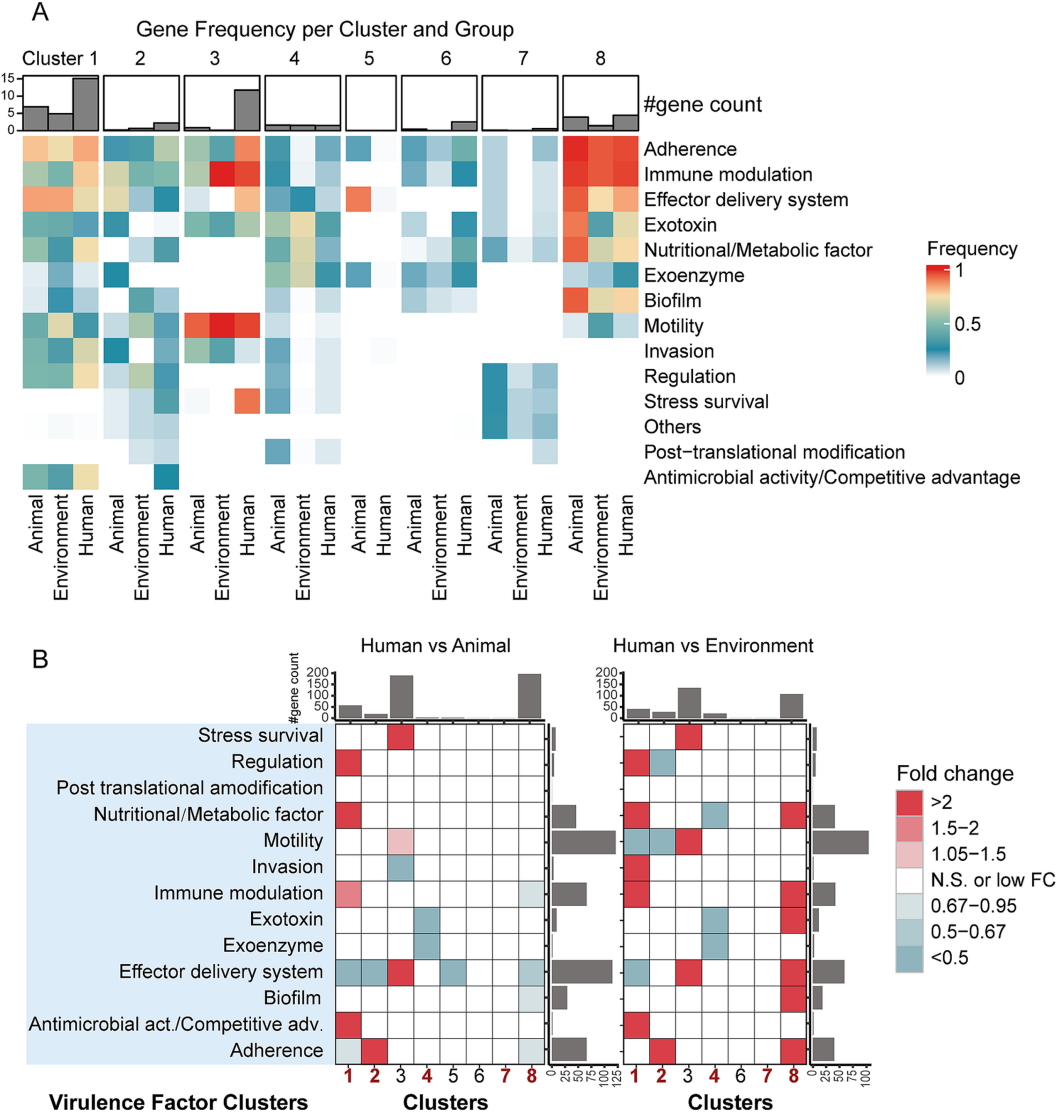
Differences in virulence factor clusters among bacterial genomes from different hosts/niches within the same taxa. **(A)** Detection rates of virulence factor clusters across groups. The heatmap color gradient represents the level of detection rates, while the bar plot above the heatmap shows the number of virulence factor-related gene types within each group. **(B)** Fold-change differences in virulence factor cluster genes between bacterial genomes from different hosts/niches within the same taxa, based on gene counts. The heatmap depicts levels of enrichment and depletion determined by the Mann–Whitney *U* test. Colored cells indicate statistically significant differences (*p* < 0.05, after FDR correction), with different colors representing varying fold changes. “N.S.” denotes non-significant differences. The bar plots on the sides represent the summed medians of gene counts for the respective row/column groups.

We then assessed the overall virulence factor load within each taxon and identified the most prevalent virulence factor clusters by calculating the average number of virulence factors in each group and determining their distribution frequency (detection rate) (Figure 3A and Supplementary Table S3). The results revealed distinct virulence factor profiles across the different taxa, closely linked to host origin. Human-derived bacteria exhibited the highest average number of virulence factors in five out of eight taxa, particularly in Cluster 1.

In general, the detection rates of virulence factors exhibit a clear host-dependent trend (Figure 3A and Supplementary Table S3): in *Pseudomonadota*, human-derived bacteria in Cluster 1 and 2, represented by *Escherichia coli* and *Legionella,* have the highest virulence factor detection rates. In contrast, in *Pseudomonadota*, Cluster 8, represented by *Pseudomonas aeruginosa*, as well as *Bacillota* (Cluster 4) and *Actinomycetota* (Cluster 7), animal-derived bacteria show the highest detection rates of virulence factors (Figure 3A and Supplementary Table S3). In *Pseudomonadota*, Cluster 1 and Cluster 8 exhibit consistent virulence factor profiles across different host ecological niches, with higher average detection rates of virulence factor clusters. In contrast, Cluster 2 has a more diverse virulence factor profile, though its detection rate is relatively lower. Specifically, in Cluster 1, human-derived bacteria show the highest average virulence factor detection rates (human: 42.08%, animal: 36.58%, environment: 36.19%), characterized by a high proportion of “Adherence” (83.4%) and “Immune modulation” (78.3%). At the same time, animal-and environmental-derived bacteria show higher detection rates of “Effector delivery system” (animal: 84.5%, environment: 84.3%). In Cluster 2, human-derived bacteria have a higher average virulence factor detection rate (human: 23.04%, animal: 18.78%, environmental: 19.82%), mainly associated with “Adherence” (83.4%) and “Immune modulation” (78.3%). Animal-derived bacteria are primarily associated with “Effector delivery system” (70.37%), while environmental bacteria have the highest detection rate for “Regulation” (60.67%). In Cluster 8, as compared to Clusters 1 and 2, animal-derived bacteria exhibit the highest average virulence factor detection rate (animal: 41.63%, human: 38.45%, environmental: 34.61%). Bacteria from all host ecological niches in this cluster frequently carry “Immune modulation” (>94%), “Adherence” (>94%), and “Effector delivery system” (>75%). In *Bacillota*-dominant Cluster 4, animal-derived bacteria have the highest average virulence factor detection rate (animal: 23.04%, environmental: 16.91%, human: 10.50%), with the most commonly detected virulence factor clusters being “Exotoxin” (>36%) and “Exoenzyme” (>30%). In Cluster 7 (within the *Actinomycetota* phylum), the overall detection rate of virulence factors is lower, with most of them being of unknown classification. The detection rate is highest in animal-derived bacteria (animal: 9.52%, human: 6.22%, environmental: 2.01%). The main virulence factor clusters in this group include “Regulation” (>6%) and “Stress survival” (>9%).

To quantify the differences in virulence gene clusters across different ecological niches, we performed a Mann–Whitney *U* test to analyze the enrichment patterns of virulence factors in each cluster, and the results were adjusted for false discovery rate (FDR) correction (Figure 3B). The results indicate that human-derived bacteria from different phyla exhibit distinct virulence factor distribution patterns: in Cluster 1, human-derived bacteria are enriched in “Regulation,” “Nutritional/Metabolic factors,” “Immune modulation,” and “Antimicrobial activity/Competitive advantage” factors, while “Effector delivery system” factors are significantly reduced. In Cluster 2, human-derived bacteria are characterized by the enrichment of “Adherence” factors. In Cluster 8, the virulence factor levels in human-derived bacteria are lower than in animal-derived bacteria but higher than in environmental-derived bacteria. In *Bacillota* (Cluster 4), human-derived bacteria show a significant reduction in “Exoenzyme” and “Exotoxin” factors, whereas in *Actinomycetota* (Cluster 7), no significant differences were observed across different sources. This virulence factor distribution pattern reflects the selective pressure of host environments on different phyla and reveals the diverse strategies pathogens use to adapt to their hosts.

In concluding, our virulence factor analysis revealed two key features: the phylum distribution characteristics of virulence factors and host-specific patterns. These features together demonstrate the diverse strategies of pathogen adaptation to the host environment and provide new perspectives for understanding host ecological niche adaptation in bacteria.

### Distribution characteristics of antibiotic resistance genes

To further investigate the relationship between host niches and pathogenic mechanisms, we utilized the CARD database to identify antibiotic resistance genes (ARGs) across 4,366 genome sequences, identifying a total of 1,343 ARGs. Among these, 123 ARGs had an occurrence frequency of over 20% across different groups, involving a variety of resistance mechanisms, with antibiotic efflux being the most prevalent. Notably, a single ARG can confer resistance to multiple antibiotics (Alcock et al., 2023), and these 1,343 ARGs were associated with 36 types of antibiotics.

**Figure 4:**
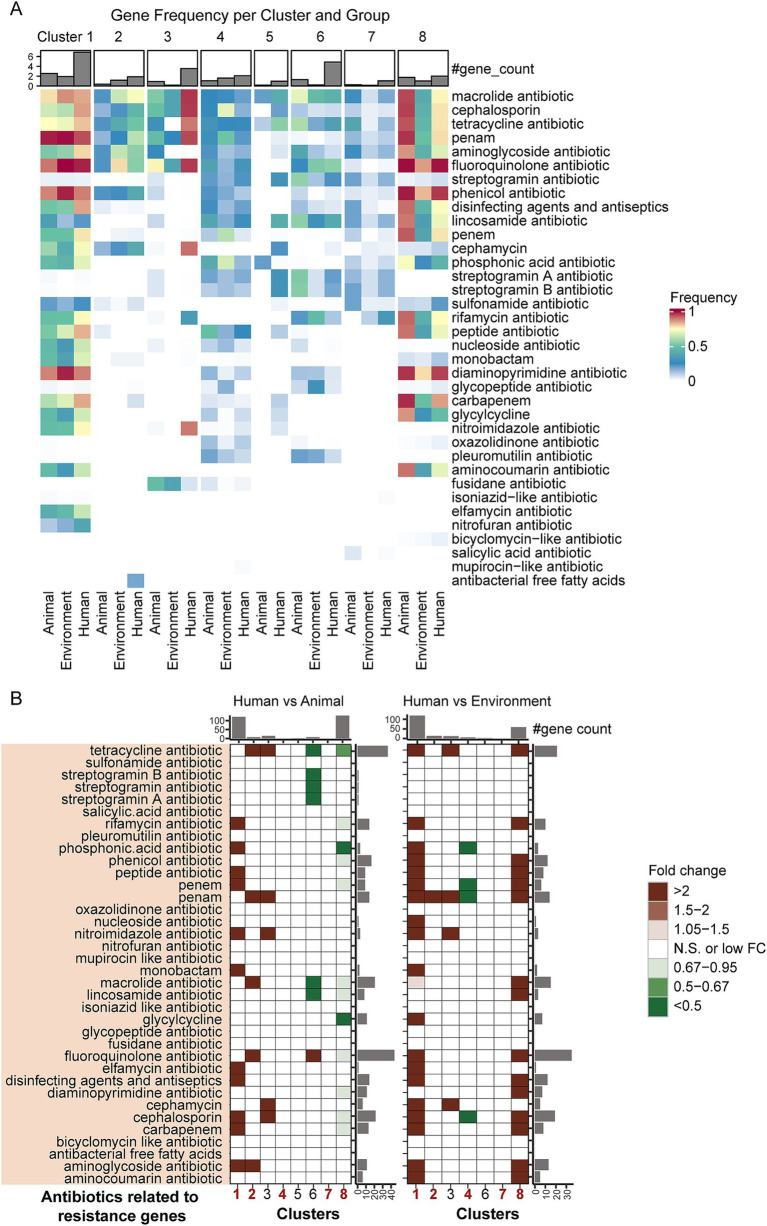
Differences in antibiotic resistance genes associated with distinct hosts/niches within the same taxa. **(A)** Detection rates of antibiotic resistance genes across groups. The color gradient in the heatmap represents varying levels of detection rates, while the bar plot above the heatmap shows the number of different types of antibiotic resistance genes detected within each group. **(B)** Fold-change differences in antibiotic resistance genes between bacterial genomes from different hosts/niches within the same taxa, based on gene counts. The heatmap illustrates levels of enrichment and depletion determined by the Mann–Whitney *U* test. Colored cells represent statistically significant differences (*p* < 0.05, after FDR correction), with distinct colors indicating different fold changes. “N.S.” denotes non-significant differences. The bar plots on the sides represent the summed medians of gene counts for the respective row/column groups.

We analyzed ARGs related to various antibiotic types across different groups to assess the overall ARG burden and potential resistance risk in these groups (Figure 4A and Supplementary Table S4). We predicted a bacterial strain to be potentially resistant to a specific antibiotic if its genome carried one or more ARGs linked to that antibiotic. The results showed that while bacteria from different sources within the same taxon had generally similar resistance profiles, there were significant differences in ARG detection rates and the average number of ARGs. Human-derived bacteria exhibited the highest potential antibiotic resistance (7/8 taxa), with the greatest average number of ARGs and the highest detection rates.

Overall, the distribution patterns of ARGs show a high degree of consistency with the previously described virulence factors (Figure 4A and Supplementary Table S4): human-derived bacteria in *Pseudomonadota* Clusters 1 and 2 (representative species being *Escherichia coli* and *Legionella*) exhibit the highest detection rates of ARGs, while animal-derived bacteria in *Pseudomonadota* Cluster 8 (represented by *Pseudomonas aeruginosa*), *Bacillota* Cluster 4, and *Actinomycetota* Cluster 7 also show relatively high detection rates of ARGs. At the phylum level, different phyla display distinct ARGs distribution patterns: in *Pseudomonadota*, Clusters 1 and 8 predominantly carry fluoroquinolone and phenicol antibiotic-related ARGs, with detection rates generally exceeding 80%, while Cluster 2 mainly harbors macrolide antibiotic-related ARGs. *Bacillota* (Cluster 4) shows a more diversified resistance profile, including peptide antibiotic (animal-derived), cephalosporin (environmental-derived), and fluoroquinolone (human-derived) ARGs. In *Actinomycetota* (Cluster 7), macrolide antibiotic-related ARGs are the most prevalent.

Moreover, human-derived bacteria exhibit unique resistance patterns across different phyla (Figure 4A and Supplementary Table S4). In *Pseudomonadota*, the ARGs in Cluster 1 human-derived bacteria are mainly associated with fluoroquinolone antibiotics (98.21%), penam (94.21%), and phenicol antibiotics (89.1%); Cluster 2 human-derived bacteria predominantly carry macrolide antibiotics (73.07%), penam (62.64%), and aminoglycoside antibiotics (55.49%) ARGs. In Cluster 8, human-derived bacteria mainly carry fluoroquinolone antibiotics (99.09%), phenicol antibiotics (96.35%), and diaminopyrimidine antibiotics (95.43%) ARGs. In *Bacillota-*dominant Cluster 4, human-derived bacteria primarily exhibit potential resistance to fluoroquinolone antibiotics (30.69%), lincosamide antibiotics (28.71%), and tetracycline antibiotics (26.73%). In *Actinomycetota* (Cluster 7), human-derived bacteria are more likely to carry rifamycin antibiotics (32.26%), macrolide antibiotics (17.74%), and lincosamide antibiotics (15.32%) ARGs.

To further explore the differences in resistance mechanisms across host sources within the same taxa, we applied the Mann–Whitney *U* test to assess the enrichment or depletion of ARGs for various antibiotic types (Figure 4B). The results revealed that, similar to the distribution of virulence genes, ARGs were most enriched in human-derived bacteria in Clusters 1 and 2. For example, in Cluster 1, human-derived bacteria showed significant enrichment of ARGs for 10 types of antibiotics, including aminoglycosides, carbapenems, cephalosporins, disinfecting agents and antiseptics, elfamycins, monobactams, nitroimidazoles, penems, peptides, and phosphonic acids. In Cluster 2, human-derived bacteria exhibited greater enrichment of penam-related resistance genes. In Cluster 8, animal-derived bacteria had significantly more ARGs than human-derived bacteria, while human-derived bacteria had more ARGs than environmental bacteria. For Cluster 4, there was no significant difference in the number of ARGs between human-and animal-derived bacteria, but human-derived bacteria showed a notable depletion of ARGs compared to environmental bacteria. In Cluster 7, no significant differences appeared in ARG distribution between host sources. These findings reveal the diversity and specificity of ARG distribution in pathogens across different host backgrounds.

By integrating the distribution patterns of antimicrobial resistance genes (ARGs) and virulence factors, we observed that these two types of pathogen-related genes exhibit highly consistent distribution patterns both at the phylum level and in terms of host origin. The co-distribution of these pathogenicity-related genes highlights the systematic regulatory strategies employed by pathogens during host adaptation, offering new insights into the ecological niche differentiation of microbial communities.

### Identification of host niches adaptation-associated signature genes

**Figure 5:**
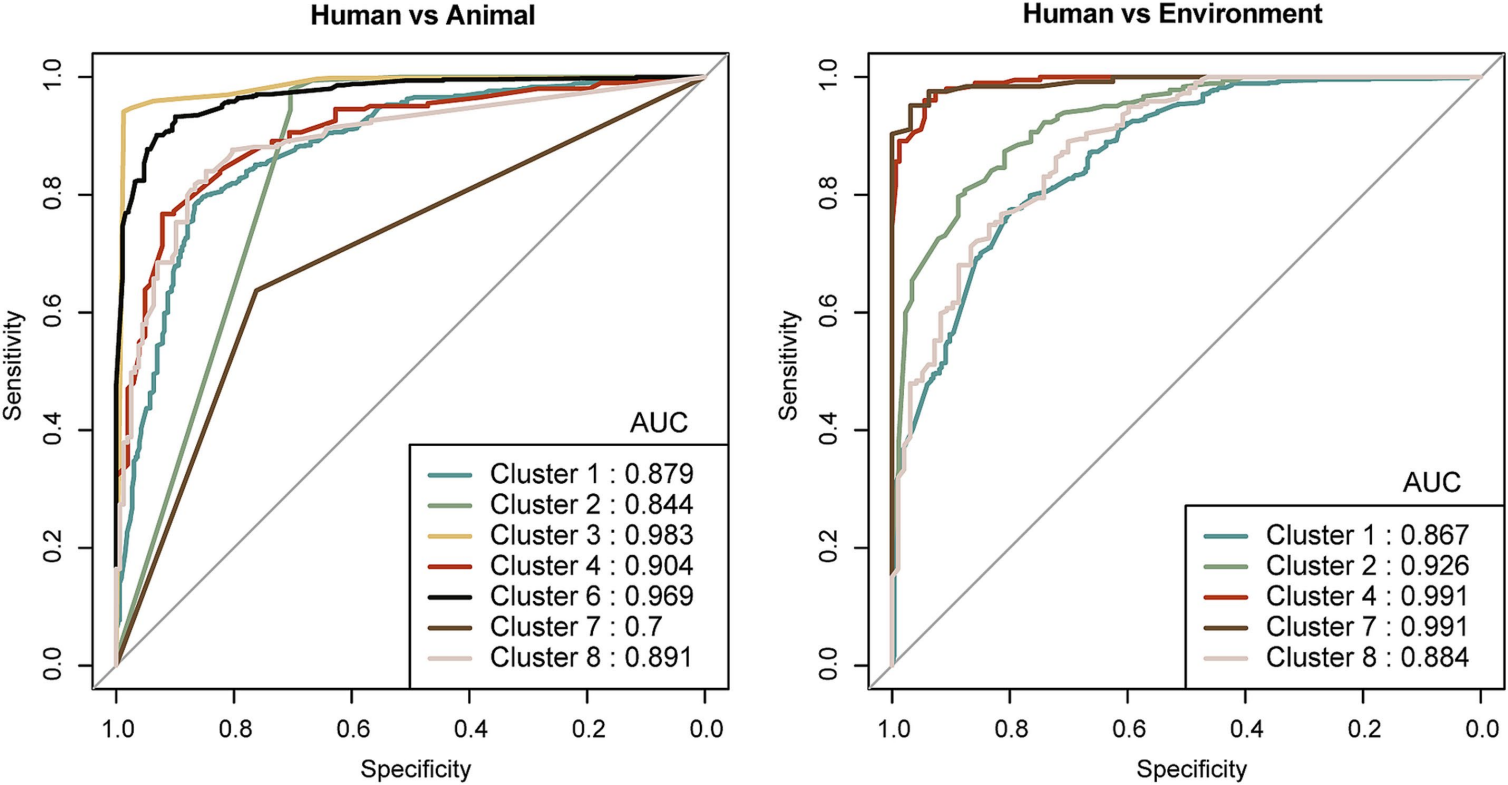
ROC curves illustrating the performance of host niches adaptation-associated signature genes identified through Scoary and machine learning approaches. The left panel shows ROC curves based on signature genes distinguishing bacteria from human hosts and animal hosts, while the right panel depicts ROC curves based on signature genes differentiating bacteria from human hosts and environmental niches. The numerical values on the plots represent the area under the curve (AUC) of the ROC curves.

To further identify host niches adaptation-associated signature genes in bacteria, we first employed Scoary for preliminary identification, as described in the Materials and Methods section. We then integrated Random Forest and LASSO models to further screen and optimize these signature genes. Finally, based on the cross-analysis results from the three methods, we plotted ROC curves and calculated AUC values to evaluate the classification performance of the selected robust signature genes. The results demonstrated that our approach effectively enhanced the model’s classification performance, though the classification effectiveness varied across different bacterial taxa (Supplementary Figure S1). Clusters 4 and 7 exhibited superior classification performance, Clusters 2 and 8 showed moderate performance, while Cluster 1 performed the worst, which may be related to niche ambiguity. The final robust signature genes selected from the classification models achieved AUC values above 0.7 and classification accuracy exceeding 0.8, indicating strong classification capability (Figure 5). This multi-layered screening method illustrated that iterative optimization improved the classification performance of signature genes, and the varying performance of different bacterial taxa in niche classification likely reflects the complexity of host-associated traits.

Through this methodology, we identified a set of signature genes closely associated with host niches (Supplementary Figure S2 and Supplementary Table S5), which demonstrated significant host bias across different taxa. These findings systematically revealed the adaptive strategies and functional characteristics of pathogens in distinct host niches. Overall, when comparing human-and animal-derived human pathogens, our study showed that the differential signature genes primarily concentrated in COG functional categories, especially in metabolism and cellular processes and signaling pathways. In terms of metabolism, the signature genes of animal-derived bacteria were mainly involved in nucleotide transport and metabolism (2/7) and secondary metabolite metabolism (3/7), while those of human-derived bacteria were related to the transport and metabolism of ions (4/11), amino acids (2/11), and carbohydrates (2/11). For cellular processes and signaling pathways, animal-derived bacteria exhibited significant enrichment in genes related to cell wall/membrane/envelope biogenesis (4/8) and post-translational modification (3/8), whereas human-derived bacteria were enriched in genes associated with post-translational modification (4/10) and intracellular secretion (2/10). Notably, two genes repeatedly appeared in cross-lineage Clusters, suggesting potential functional conservation across hosts. For example, the COG0378 (*hypB*) gene cluster was significantly enriched in human-derived bacteria from Clusters 1 and 4, and the COG1704 (*LemA*) gene cluster was enriched in animal-derived bacteria from Clusters 1 and 7. This observation suggests potential functional conservation of these genes across host species.

On the other hand, environmental bacteria exhibited significant enrichment in carbohydrate-active enzyme (CAZyme) genes (11/58), and their metabolic signature genes were relatively abundant (16/58). Additionally, in pathways related to “Information Storage and Processing,” transcription-related genes represented a substantial proportion (4/5) in environmental bacteria. In contrast, human-derived bacteria exhibited enrichment in “Information Storage and Processing” pathways (8/18), primarily involving translation-related genes (5/8). Overall, environmental bacteria demonstrated a more diverse functional profile, particularly in their ability to metabolize various carbon sources via carbohydrate-active enzymes, thereby adapting to the survival demands of complex environments.

## Discussion

There is growing recognition that host-microbe interactions play a critical role in health and disease. Understanding host-pathogen relationships at the genomic level could enable researchers to develop targeted strategies for infection control and prevention. However, most studies have focused on individual pathogen species or microbial communities, placing more emphasis on microbial diversity rather than gene function (Sheppard et al., 2013; Wheeler et al., 2018; Zhou et al., 2024). In this study, our primary goal was to explore the adaptive differences of bacterial pathogens across distinct ecological environments. By analyzing 4,366 high-quality, non-redundant genome sequences of human pathogenic bacteria classified into three host ecological niches—human, non-human animals, and environment—we aimed to identify genetic adaptation patterns associated with different hosts or environments. This approach provides insights into potential mechanisms underlying cross-host transmission and environmental dissemination of pathogens.

Our analysis reveals the adaptive traits of these microorganisms in terms of functional genes, virulence factors, and antibiotic resistance genes. The results indicate a complex and intimate association between the genomic characteristics of pathogens and their host niches, reflecting the pathogens’ adaptation to diverse environments over the course of long-term evolution. Bacterial clusters show specific adaptations to their ecological niches, highlighting the multi-level strategies employed to thrive in human, animal, and environmental contexts.

The phylum *Pseudomonadota*, particularly members residing in the human gut, has shown evidence of extensive co-evolution with humans. Compared to non-human hosts, bacteria isolated from human hosts exhibit significant enrichment in COG functional category genes and carbohydrate enzyme genes. This may relate to higher horizontal gene transfer observed in human-associated bacteria, increasing genetic diversity and adaptation in humans (Smillie et al., 2011). Meanwhile, *Pseudomonadota* also display higher detection rates of adhesion factors and immune-modulating factors, with studies suggesting that adhesion and immune evasion mechanisms are key strategies for bacterial colonization in humans (Kelly et al., 2005).

Further analysis revealed that bacteria in Cluster 1 (such as *Enterobacter*, *Escherichia*, and *Vibrio*) are widely present in the nutrient-rich human gut, with rapid growth and efficient protein synthesis (Dethlefsen and Schmidt, 2007; Vieira-Silva and Rocha, 2010). In contrast, Cluster 2 and Cluster 8 (including *Stenotrophomonas* spp., *Burkholderia* spp., *Legionella* spp., *Acinetobacter* spp., and *Pseudomonas* spp.) demonstrate broader niche adaptability, being not only isolated from clinical samples but also widely distributed across various environments such as soil, water bodies, and artificial water systems. These bacteria exhibit exceptional environmental adaptability and metabolic diversity, enabling them to utilize a wide range of nutrient sources for growth and reproduction (Atlas, 1999; Compant et al., 2008; Ryan et al., 2009; Silby et al., 2011; Wong et al., 2017). Notably, human-derived bacteria from Cluster 1 and Cluster 2 display a higher detection rate of antibiotic resistance genes (ARGs), likely due to frequent exposure to antibiotics in clinical settings (Berglund, 2015). In contrast, animal-derived bacteria in Cluster 8 exhibit higher virulence factor and ARG loads, further emphasizing the role of animal hosts as important reservoirs for ARGs (Hu et al., 2017).

We also found that environmental bacteria (such as *Bacillus* and *Staphylococcus* in Cluster 4 and *Actinomycetota* in Cluster 7) exhibit greater enrichment of genes related to metabolism and transcriptional regulation compared to host-derived bacteria. This suggests that these bacteria require more flexible metabolic and regulatory strategies to adapt to environmental survival pressures in complex and dynamic conditions (Balasubramanian et al., 2017; Borriss, 2020). Exotoxins and exoenzymes are the most prevalent virulence factors in Cluster 4, primarily distributed among environmental bacteria, while the virulence factor and antibiotic resistance gene burdens in *Actinomycetota* are relatively limited. The limited identification of virulence and resistance factors in *Actinobacteria* may be due to the restricted number of genomes included in this study and the fact that we performed gene-level identification. Future analyses at the SNP level will be crucial for further exploring host-specific genes in key pathogens within this phylum.

Additionally, we observed that fluoroquinolone-related resistance genes were the most widely distributed across the samples. This finding suggests that the widespread use of fluoroquinolone antibiotics may have driven the pervasive dissemination of these resistance genes in both environmental and human-associated bacteria, facilitated by horizontal gene transfer (HGT) among different strains (Redgrave et al., 2014). This distribution complicates treatment and poses challenges. Therefore, it is necessary to further monitor the transmission pathways of these genes and their potential impact on bacterial ecological adaptation, in order to develop more effective antibiotic usage strategies and resistance control measures.

We identified a set of signature genes closely associated with host niches, spanning multiple functional categories such as metabolism, signal transduction, and transcriptional regulation. A particularly notable finding is the presence of the *hypB* gene across multiple human-associated bacterial taxa. *hypB* encodes a GTP-binding protein that plays a critical role in nickel transport and hydrogenase enzyme maturation, both of which are essential for bacterial energy metabolism (Fu et al., 1995; Yang et al., 2022). In addition, its potential involvement in modulating host immune responses suggests that *hypB* may serve as a key determinant of bacterial adaptation to the human host (Lacasse et al., 2019; Morrison et al., 1990). This highlights its importance as a target for further functional studies.

Despite these findings, there are certain limitations to this study that warrant consideration. First, our analysis relies on the metadata available for bacterial genomes, particularly their isolation source, to define ecological niches. This approach may oversimplify the ecological and biological complexity of pathogen-host interactions, as some bacteria may transition between different niches or display overlapping adaptive traits. For example, *Campylobacter jejuni* colonizes poultry and infects humans (Mouftah et al., 2021), while *Staphylococcus aureus* transitions between livestock and human hosts (Weinert et al., 2012). Additionally, while we categorized host niches into human, non-human animal, and environment, the non-human animal category encompasses diverse ecological contexts (e.g., infection-related and commensal samples), which may require more detailed stratification in future studies. Moreover, metadata variability and inconsistencies could introduce biases in niche classification, limiting the granularity of our findings. Future studies should aim to refine ecological niche classifications, particularly by incorporating more detailed metadata and accounting for overlapping adaptive traits across host and environmental contexts.

In conclusion, large-scale comparative genomics was utilized in this study to uncover the adaptive strategies and evolutionary mechanisms of human pathogens within various host niches. By demonstrating how host niches shape the distribution of genomic functions, virulence factors, and antibiotic resistance genes in these pathogens, our findings not only deepen the understanding of pathogen adaptation but also offer a critical theoretical basis for developing targeted infection control strategies and improving antibiotic stewardship.

## Data Availability

The original contributions presented in the study are included in the article/Supplementary material, further inquiries can be directed to the corresponding author.

## Footnotes

^1^ https://nmdc.cn/gcpathogen/

^2^ https://itol.embl.de/

## Supplementary material

SUPPLEMENTARY FIGURE S1 PCoA analysis of gene cluster contributions to host niches differentiation in bacterial genomes. **(A–C)** Represent different taxa. We visualized the overall contribution of statistically significant enriched/depleted gene clusters to the differentiation of bacterial genomes from distinct hosts/niches using principal coordinates analysis (PCoA). The analysis was performed using Canberra distance on two types of genome-wide matrices: (1) the full matrix containing all gene clusters annotated from various databases within a taxon, and (2) the matrix containing only the gene clusters identified as enriched/depleted through different methods (Scoary, Random Forest, LASSO). Blank plots indicate that the method was not suitable for identifying host niches adaptation-associated signature genes in that group. Each scatter plot includes the Akaike information criterion (AIC) value from logistic regression fitting, with lower AIC values indicating better model fit to the data.

SUPPLEMENTARY FIGURE S2 Venn diagram illustrating the number of important feature gene clusters predicted by Random Forest and LASSO. The numbers represent gene clusters identified exclusively in each method (non-overlapping regions) or shared between the two methods (overlapping regions). Pink represents gene clusters identified by Random Forest, while yellow represents those identified by LASSO.

SUPPLEMENTARY TABLE S1 Metadata summary of 4,366 bacterial genome sequences used in this study.

SUPPLEMENTARY TABLE S2 Description of COG categories.

SUPPLEMENTARY TABLE S3 Detection rates of virulence factor clusters across different groups.

SUPPLEMENTARY TABLE S4 Detection rates of antibiotic resistance genes across different groups.

SUPPLEMENTARY TABLE S5 List of host niches adaptation-associated signature genes identified by different methods.
