# Supplementary figures and images for: Comparative genomics reveals key adaptive mechanisms in pathogen host-niche specialization
Source: Front Microbiol. 2025 Jun 6;16:1543610. doi: 10.3389/fmicb.2025.1543610 (PMC12180303; doi:10.3389/fmicb.2025.1543610)

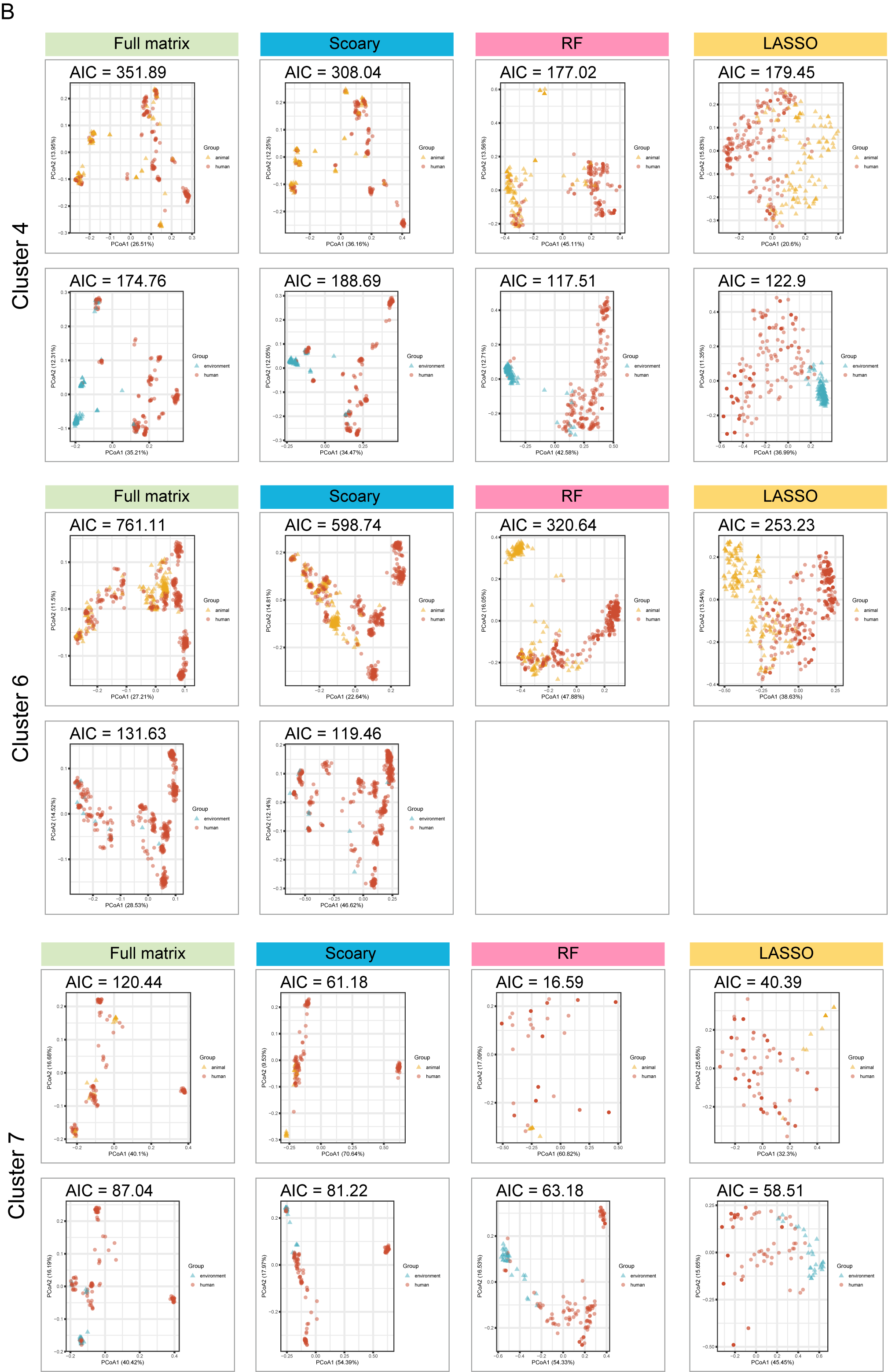

Supplement: SUPPLEMENTARY FIGURE S1 — PCoA analysis of gene cluster contributions to host niches differentiation in bacterial genomes. (A–C) Represent different taxa. We visualized the overall contribution of statistically significant enriched/depleted gene clusters to the differentiation of bacterial genomes from distinct hosts/niches using principal coordinates analysis (PCoA). The analysis was performed using Canberra distance on two types of genome-wide matrices: (1) the full matrix containing all gene clusters annotated from various databases within a taxon, and (2) the matrix containing only the gene clusters identified as enriched/depleted through different methods (Scoary, Random Forest, LASSO). Blank plots indicate that the method was not suitable for identifying host niches adaptation-associated signature genes in that group. Each scatter plot includes the Akaike information criterion (AIC) value from logistic regression fitting, with lower AIC values indicating better model fit to the data. [file Supplementary_file_1.zip › Supplementary file 1B.TIF]

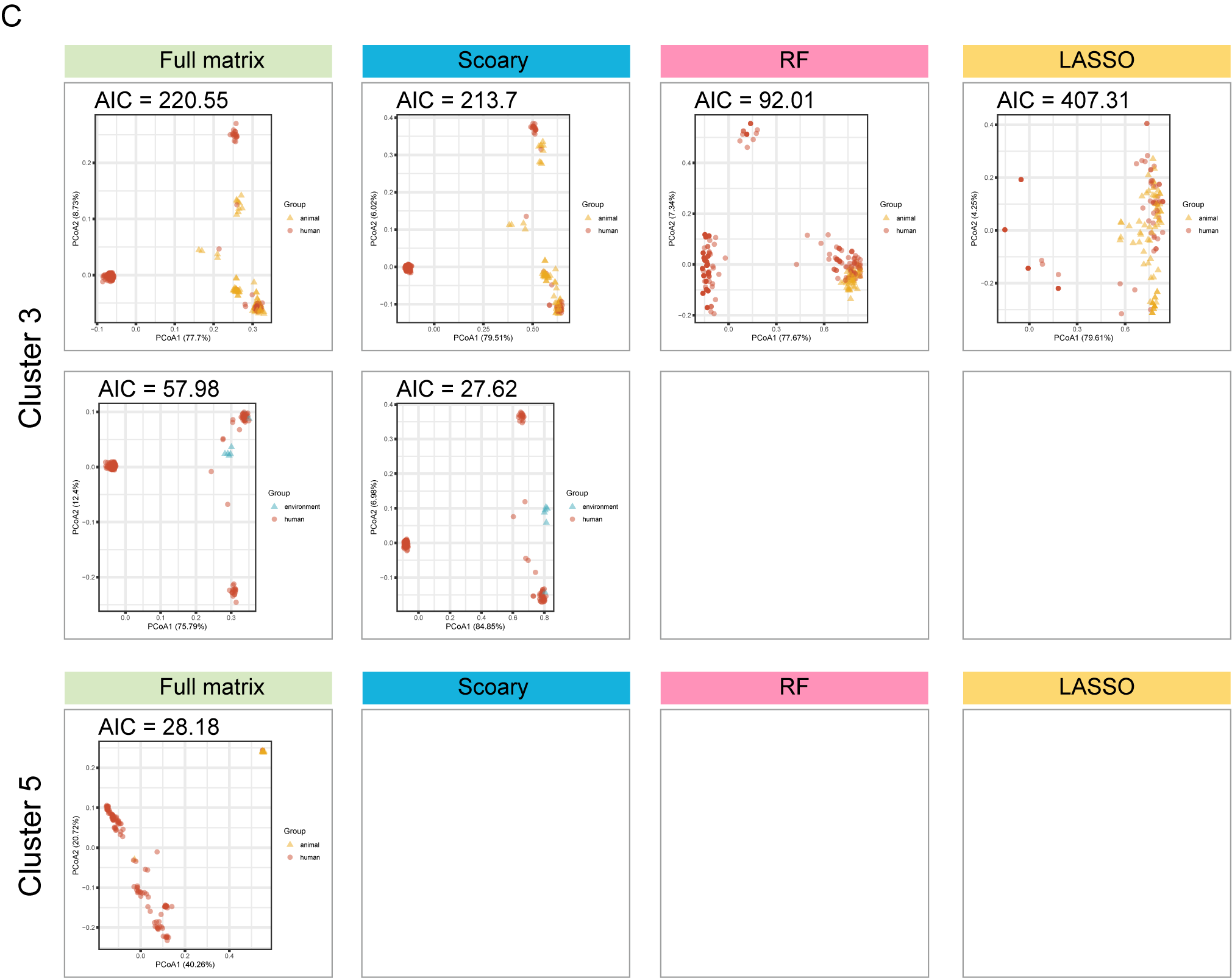

Supplement: SUPPLEMENTARY FIGURE S1 — PCoA analysis of gene cluster contributions to host niches differentiation in bacterial genomes. (A–C) Represent different taxa. We visualized the overall contribution of statistically significant enriched/depleted gene clusters to the differentiation of bacterial genomes from distinct hosts/niches using principal coordinates analysis (PCoA). The analysis was performed using Canberra distance on two types of genome-wide matrices: (1) the full matrix containing all gene clusters annotated from various databases within a taxon, and (2) the matrix containing only the gene clusters identified as enriched/depleted through different methods (Scoary, Random Forest, LASSO). Blank plots indicate that the method was not suitable for identifying host niches adaptation-associated signature genes in that group. Each scatter plot includes the Akaike information criterion (AIC) value from logistic regression fitting, with lower AIC values indicating better model fit to the data. [file Supplementary_file_1.zip › Supplementary file 1C.TIF]

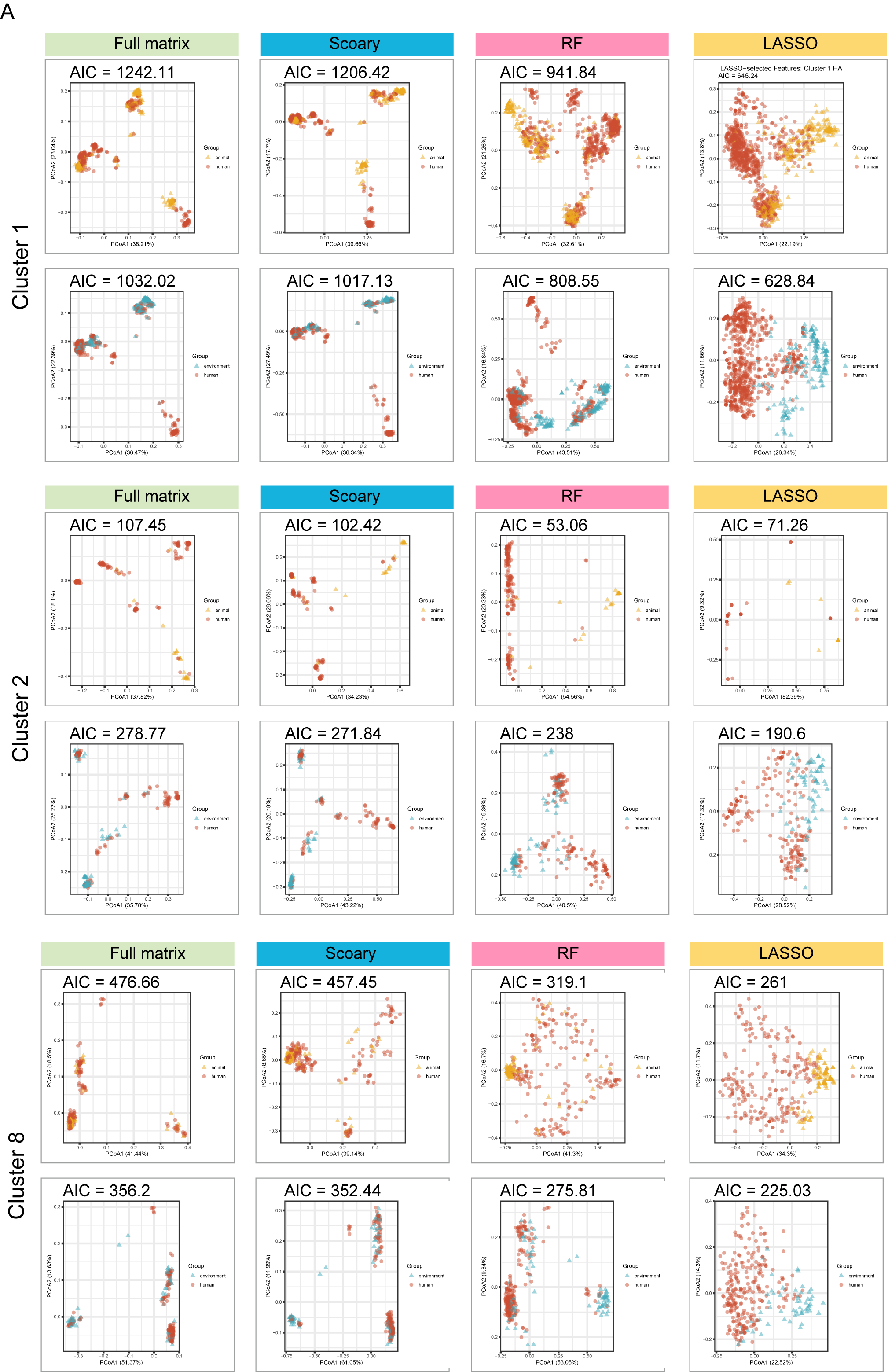

Supplement: SUPPLEMENTARY FIGURE S1 — PCoA analysis of gene cluster contributions to host niches differentiation in bacterial genomes. (A–C) Represent different taxa. We visualized the overall contribution of statistically significant enriched/depleted gene clusters to the differentiation of bacterial genomes from distinct hosts/niches using principal coordinates analysis (PCoA). The analysis was performed using Canberra distance on two types of genome-wide matrices: (1) the full matrix containing all gene clusters annotated from various databases within a taxon, and (2) the matrix containing only the gene clusters identified as enriched/depleted through different methods (Scoary, Random Forest, LASSO). Blank plots indicate that the method was not suitable for identifying host niches adaptation-associated signature genes in that group. Each scatter plot includes the Akaike information criterion (AIC) value from logistic regression fitting, with lower AIC values indicating better model fit to the data. [file Supplementary_file_1.zip › Supplementary file 1A.TIF]

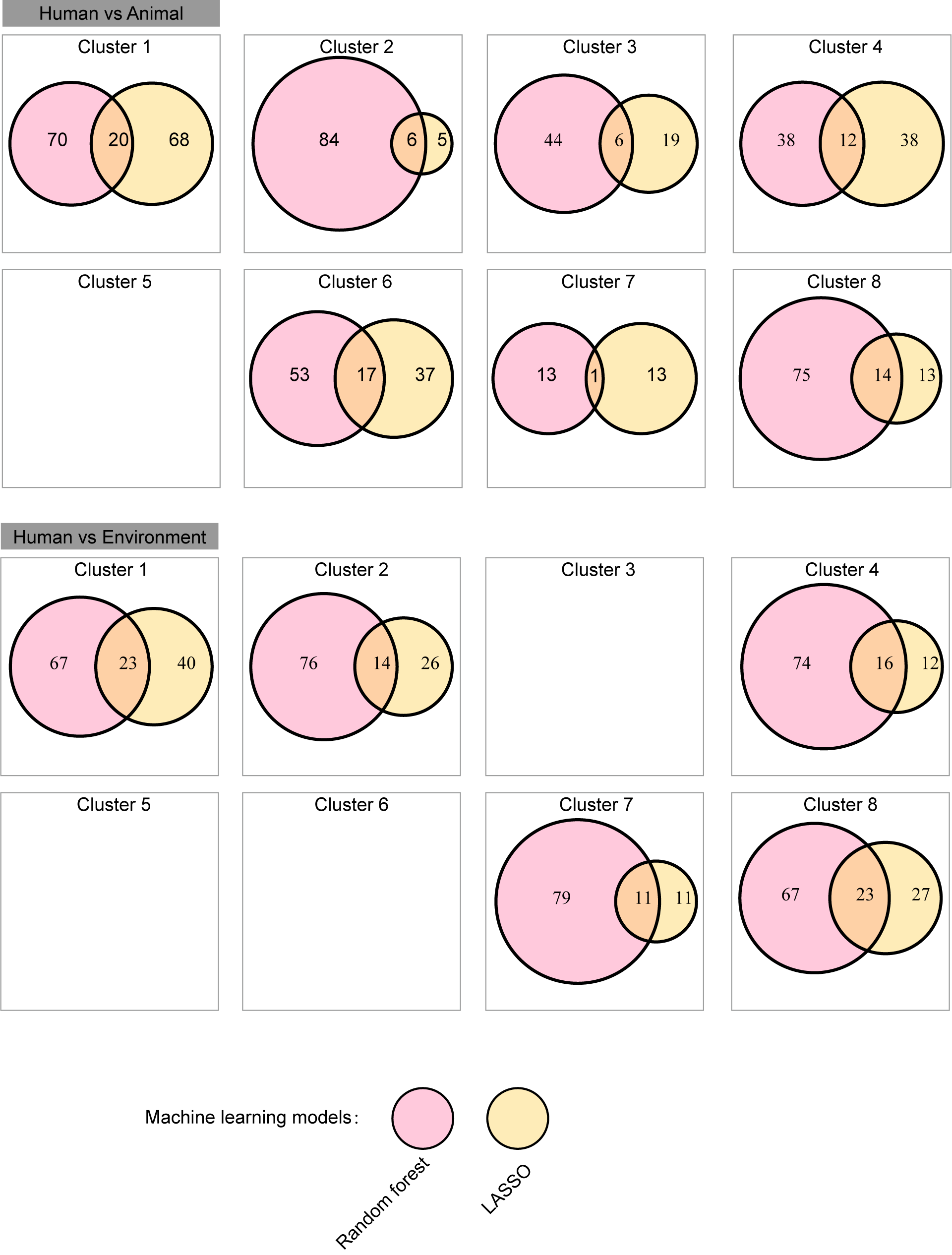

Supplement: SUPPLEMENTARY FIGURE S2 — Venn diagram illustrating the number of important feature gene clusters predicted by Random Forest and LASSO. The numbers represent gene clusters identified exclusively in each method (non-overlapping regions) or shared between the two methods (overlapping regions). Pink represents gene clusters identified by Random Forest, while yellow represents those identified by LASSO. [file Supplementary_file_2.tif]
